# Preterm birth does not increase the risk of developmental dysplasia of the Hip: a systematic review and meta-analysis

**DOI:** 10.1186/s12887-023-04083-1

**Published:** 2023-05-29

**Authors:** Amirhossein Ghaseminejad-Raeini, Parmida Shahbazi, Ghazale Roozbahani, Amirmohammad Sharafi, Seyyed Hossein Shafiei, Yousof Fallah, Soroush Baghdadi

**Affiliations:** 1 Orthopedic Surgery Research Center (OSRC), Sina University Hospital, Tehran University of Medical Sciences, Tehran, Iran; 2 Division of Orthopaedics, The Children’s Hospital of Philadelphia, Philadelphia, PA USA

**Keywords:** Developmental dysplasia of the Hip, Preterm birth, Congenital, Dysplasia

## Abstract

**Background:**

The purpose of this systematic review was to appraise the literature on the association between preterm birth and developmental dysplasia of the hip (DDH).

**Methods:**

Medline, Embase, Scopus, and Web of Science databases were queried for all studies pertaining to DDH and preterm birth. Data were imported and analyzed in Revman5 and Comprehensive Meta-Analysis (CMA) for pooled prevalence estimation.

**Results:**

Fifteen studies were included in the final analysis. There were 759 newborns diagnosed with DDH in these studies. DDH was diagnosed in 2.0% [95%CI:1.1–3.5%] of the premature newborns. Pooled incidence rate of DDH was not statistically different between those groups (2.5%[0.9%-6.8%] vs. 0.7%[0.2%-2.5%] vs. 1.7%[0.6%-5.3%];Q = 2.363,*p* = 0.307).

**Conclusions:**

In this systematic review and meta-analysis, we did not find preterm birth to be a significant risk factor for DDH. Data suggests that female sex and breech presentation are associated with DDH in preterm infants, but the data is scarce in the literature.

**Supplementary Information:**

The online version contains supplementary material available at 10.1186/s12887-023-04083-1.

## Background

Developmental dysplasia of the hip (DDH) is one of the most common orthopaedic disorders of childhood, with an estimated incidence of 1–10 per 1,000 live births [1]. DDH presents as a spectrum, ranging from mild dysplasia to high-riding hip dislocation [2]. Symptoms are generally absent until later in life, with patients presenting in childhood with limping and leg length discrepancy, to young adulthood with degenerative changes [3, 4]. Considering the long-term consequences of untreated DDH, and the asymptomatic nature of early disease, different screening protocols are utilized throughout the world, depending on the incidence and healthcare resources availability, among other factors. This may include a clinical examination by the primary care team, ultrasound screening in high-risk patients, to universal ultrasound screening of all neonates [1, 5–7].

Risk factors of DDH have been studied extensively. Breech presentation, female sex, firstborn status, and positive family history are believed to be the most important risk factors [8]. Preterm birth is defined as birth before 37 completed weeks of pregnancy. Globally, about 11% of pregnancies result in a preterm birth, and 15 million preterm infants are born every year [9]. Musculoskeletal complications of preterm birth include fractures, metabolic bone disease, and cerebral palsy [10]. While there have been speculations regarding the association between prematurity and DDH, the literature is inconclusive [11, 12]. Therefore, we performed this study to systematically review and meta-analyze the literature regarding the association between preterm birth and DDH. We hypothesized that we would not find evidence supporting an association between preterm birth and DDH.

## Methods

This study was conducted following the Preferred Reporting Items for Systematic Reviews and Meta-Analyses (PRISMA) guidelines. Prior to the initiation of this study on 5^th^ October 2022, our protocol was registered in the International prospective register of systematic reviews (PROSPERO) under the registration code: CRD42022357984.

### Eligibility criteria

Studies were eligible for inclusion if they reported on the association between preterm birth and DDH. Prospective and retrospective cohort studies and cross-sectional studies were eligible. Studies with unavailable full text, incomplete data, systematic reviews, case series, case reports, pilot studies, letters, correspondents, and commentaries and non-English publications were excluded.

### Search strategy

A systematic literature review was conducted on 1^st^ September 2022 on the following electronic databases: Medline, Embase, Scopus, and Web of Science without any restrictions on publication date. Derived from our research question, "Is there an association between preterm birth and DDH” an expanded search based on Medical Subject Headings (MeSH) and keywords with Boolean operators ("Developmental Dysplasia of the Hip" [Mesh]; "Developmental Hip Dysplasia*"; "Developmental Hip Dislocation*"; DDH; "Premature Birth" [Mesh]; "Preterm Birth*"; "Birth Preterm"; "Birth Premature"; "Preterm Neonate*"; "Preterm"; "Premature Infant*"; "Preterm Infant*"; "Premature") was performed and documented in Supplementary file 1. In addition, a manual search was performed on 10^th^ December 2022 to identify more studies trough a snowballing technique.

### Selection process

Two independent reviewers (P.Sh and Gh.R) screened the search results using Covidence systematic review management software and in cases of any disagreement, a third author (A.Gh) supervised the process and made the final decision.

### Data extraction

Dual independent data extraction was conducted by two authors (P.Sh and Gh.R) and discrepancies were discussed and resolved by the third author (A.Gh). Basic data including: the first author, publication year, country of origin for the study, study design, study center and the number of centers/clinics/areas, sample size, sex (Girl: boy), delivery type (cesarian or normal vaginal delivery (NVD)), birth weight in grams, breech presentation, and family history of DDH were recorded. Preterm and term population data consist of: Number of terms and preterm cases, preterm and term definition, inclusion and exclusion criteria, and loss to follow-up. And the outcome data extraction involves the age of DDH diagnosis, screening methods, types of Graf classification, number of patients with DDH in term and preterm children, and number of hips with DDH in term and preterm children. If the studies include the number of hips with DDH except for the number of participants with DDH, we will contact the study authors to obtain data on the number of cases with DDH. Extracted data imported to an excel sheet for further analyses and synthesis.

### Quality assessment

Included studies were assessed independently by the same authors for possible reporting biases by Risk Of Bias In Non-randomized Studies—of Interventions (ROBINS-I), a 7-items checklist [13]. All disagreements were discussed and resolved by the third reviewer.

### Statistical analysis

#### Assessment of heterogeneity

To test heterogeneity the Chi^2^ test was used for statistical significance and the I^2^ statistic was applied to quantify heterogeneity. The heterogeneity degree was graded as 0% to 30%, which might not be important; 31% to 50%, moderate heterogeneity; 51% to 75%, substantial heterogeneity; 76% to 100%, considerable heterogeneity. If heterogeneity was found, potential reasons were explored and performed subgroup analyses based on gestational age (≤ 37 weeks and > 37 weeks), type of Graft classification, region (Australia, East Asia, Europe, Middle East, and North America), risk factors for DDH including sex (Girl: boy), breech presentation, and oligohydramnios.

#### Data synthesis

Data were imported and analyzed in Revman 5 for comparative analyses and Comprehensive Meta-Analysis (CMA) for pooled prevalence estimation. In case of homogeneity, fixed-effect model was planned to pool results and obtain the fixed-effect RR, weighted MD (WMD), and standardized mean different (SMD), where appropriate. If heterogeneity was found and data were thought to be suitable to pool, then a random-effects model was used.

### Publication bias

The publication bias was assessed by examining the degree of asymmetry of a funnel plot in RevMan 5.4 and Egger’s regression test in CMA.

## Results

### Study characteristics and quality assessment

**Fig. 1:**
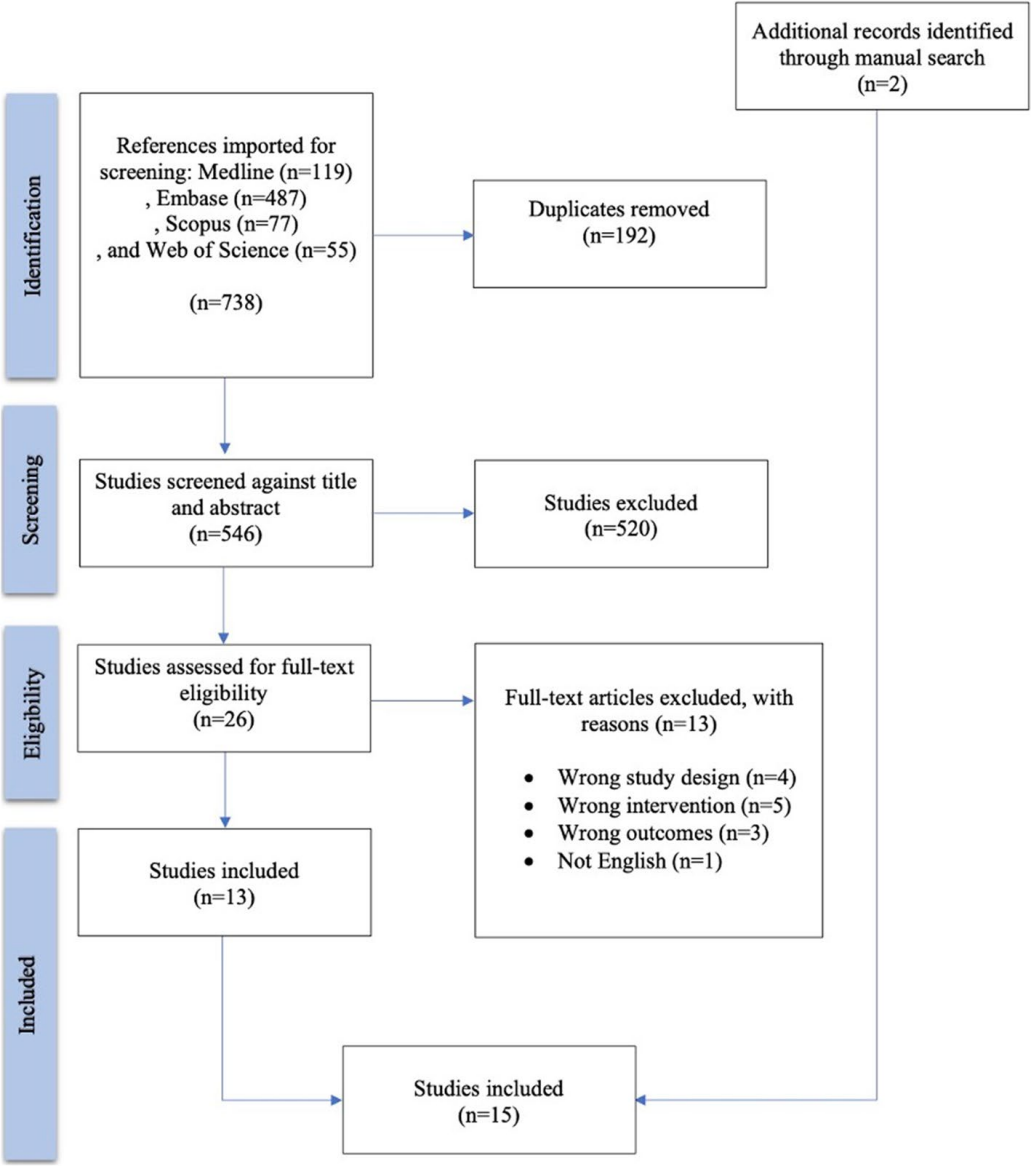
PRISMA flowchart of the present systematic review

Among a total of 738 references that were imported for screening, 192 duplicates were removed and 546 studies remained for screening against title and abstract. 26 studies assessed for full-text eligibility. Another 13 studies were excluded in the full-text review: ineligible study design (*n* = 4), wrong intervention (*n* = 5), wrong outcomes (*n* = 3), and not English (*n* = 1). Two studies were identified through manual research and finally, 15 studies were included in this systematic review (Fig. 1) [6, 8, 11, 12, 14–24]. Risk of bias assessment is summarized in Table 1. One third of the included studies were prospectively designed [8, 12, 17, 20, 23]. Six, Seven, and two articles were deemed to have low, moderate, and high risk of bias, respectively. Risk of Selection bias and bias due to deviations from intended interventions were two major reasons that made us to classify two studies in the high-risk group [12, 16]. The total population comprised of 35,030 infants, of whom approximately 51.4% were girls and 20.0% were premature. Delivery types were discussed in 6 articles (21,372 infants), which was a Caesarean section in 46.8% [6, 15, 19, 21, 22, 24]. Based on 11 out of 15 studies, 14.6% had a breech presentation at the time of delivery. Main characteristics of the eligible articles are summarized in Table 2 [6, 12, 14, 15, 17–19, 21–24].

**Table 1:** Studies risk of bias based on ROBINS-I tool for non-interventional studies

Study, year	Study design	Risk of selection bias	Risk of bias in classification of interventions	Risk of bias due to deviations from intended interventions	Risk of attrition bias	Risk of outcome measurement bias	Risk of bias from confounding factors	Risk of statistical analysis bias	Overall risk of bias
Gardiner et al., 1990 [14]	Retrospective	Moderate	Moderate	Low	Unclear	Low	Unclear	Low	Moderate
Xu et al., 2022 [24]	Retrospective	Low	Low	Low	Low	Low	Moderate	Moderate	Moderate
Hegde et al., 2020 [15]	Retrospective	Low	Low	Low	Low	Low	Moderate	Low	Low
Koob et al., 2022 [16]	Retrospective	High	Low	High	Unclear	Moderate	Unclear	Moderate	High
Lange et al., 2017 [17]	Prospective	Low	Low	Low	Low	Low	Low	Low	Low
Orak et al., 2015 [20]	Prospective	Moderate	Low	Low	Low	Low	Moderate	Low	Low
Quan et al., 2013 [22]	Retrospective	Moderate	Moderate	Low	Unclear	Moderate	Unclear	Low	Moderate
Pulik et al.,2022 [25]	Retrospective	Low	Low	Low	Low	Low	Low	Low	Low
Lee et al., 2016 [18]	Retrospective	Low	Low	Unclear	Low	Low	Unclear	Low	Moderate
Sezer et al., 2013 [8]	Prospective	Low	Low	Unclear	Low	Low	Unclear	Low	Low
Duramaz et al.,2019 [11]	Retrospective	Low	Low	Unclear	Low	Low	Unclear	Low	Low
Jeon et al.,2022 [6]	Retrospective	Low	Low	Unclear	Low	Moderate	Moderate	Low	Moderate
Leonard et al.,2022 [19]	Retrospective	Low	Moderate	Unclear	Low	Low	Unclear	Low	Moderate
Simić et al., 2009 [23]	Prospective	Moderate	Moderate	Moderate	Unclear	Low	Unclear	Moderate	Moderate
Tuncay et al.,2005 [12]	Prospective^a^	High	Low	Moderate	Low	Low	Unclear	Low	High

^a^Unclear due to lack of direct information

**Table 2:** Major characteristics of the included studies. NVD: normal vaginal delivery, CS: Caesarean section, DDH: developmental dysplasia of the hip

Study, year	Country	Number of centers/clinics/areas	Participants (N)	Girl: Boy	Delivery type		Birth weight (Mean grams ± SD)	Breech presentation (N)	Positive ddh family history (N)
					NVD	CS			
**Gardiner et al., 1990 **[14]	England	2	164	69:95	_*	_	_	37	16
**Xu et al., 2022 **[24]	Chinese	Multi-center	19,833	10,881:8952	9134	6757	_	1871	18
**Hegde et al., 2020 **[15]	Australia	1	1144	637:507	227	917	Median, (IQR) (range) 23–27 weeks 830 (686–980) (420–1375) ______ 28–31 weeks 1338 (1134–1560) (600–2280) _______ 32–36 weeks 2165 (1860–2448) (950–4495) _______ ≥ 37 weeks 3073 (2759–3555) (1870–5135)	1144	_
**Koob et al., 2022 **[16]	Germany	2	660	Error	_	_	_	_	E^**^
**Lange et al., 2017 **[17]	Germany	Multi-center	2910	1394:1513	_	_	Term: 3463 ± 480 Preterm: 2067 ± 724	230	181
**Orak et al., 2015 **[20]	Turkey	1	467	206:261	_	_	_	E	E
**Quan et al., 2013 **[22]	Australia	1	292	143:149	42	250	reported in just 6 DDH term and preterm patients: 1365, 2350, 3030, 4170, 3500, 3965 g	292	reported 0 in DDH patients (both term and preterm)
**Pulik et al., 2022 **[25]	Poland	1	3102	1541:1561	1240	1262	Median (Q1–Q3) 3.40 (3.09–3.73)	173	284
**Lee et al., 2016** [18]	USA	1	318	164:154	_	_	< 32 weeks: 1158 ± 414 32–37 weeks: 2070 ± 440	318	_
**Sezer et al., 2013 **[8]	Turkey	1	421	206:215	_	_	1401.4 ± 366.7	Error	E
**Duramaz et al., 2019 **[11]	Turkey	1	394	208:186	_	_	2031 ± 495	E	E
**Jeon et al., 2022 **[6]	Korea	1	155	84:71	2 DDH patients	8 DDH patients	DDH patients: 1240 ± 237 Normal:1295 ± 335	48	_
**Leonard et al., 2022** [19]	USA	1	1533	_	718	815	1722 ± 611	428	_
**Simić et al., 2009** [23]	Serbia	1	2045	904:1141	_	_	2067.1	183	_
**Tuncay et al., 2005** [12]	Turkey	1	1592	837:755	_	_	_	115	18

### DDH incidence in preterm infants

**Fig. 2:**
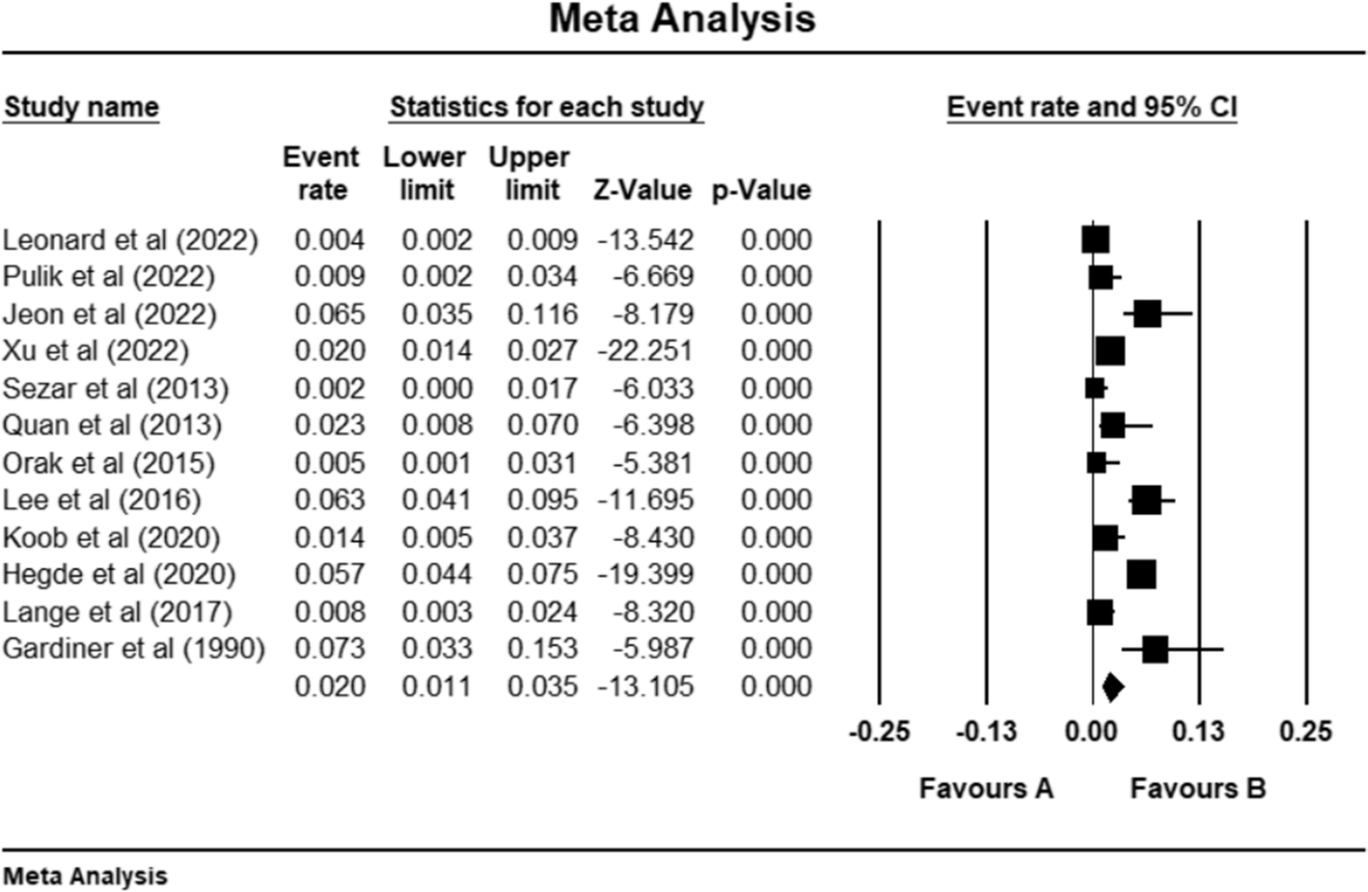
Pooled prevalence of DDH in preterm infants

There were 759 newborns diagnosed with DDH in the included studies (Table 3). Pooled incidence of DDH in preterm children was calculated utilizing data from 12 studies [6, 8, 14–22, 24]. According to the meta-analysis, DDH was diagnosed in 2.0% [95% CI: 1.1—3.5%] of the premature newborns (Fig. 2). However, there was considerable data heterogeneity of the studies (Q = 94.55, I^2^ = 88.36%, *p* < 0.001). Therefore, we performed subgroup analyses in order to detect significant differences between subgroups regarding the preterm definition, DDH Graf type, and study region. Eight studies set a limit of 37 weeks to define preterm birth [8, 14–18, 21, 22], but lower than 37 weeks in three other studies [6, 19, 20]. However, there was no significant difference between groups in terms of preterm birth definition (2.5% [1.3%—4.6%] vs. 1.1% [0.1%—9.6%]; Q (subgroup difference) = 0.454, *p* = 0.500) (Supplementary file 2). Also, the definition of DDH was not identical in the studies. Graf types IIa and above, IIb and above, or IIc and above were considered DDH in three [18, 21, 24], two [16, 19], and five articles [8, 14, 15, 17, 20], respectively. Pooled incidence rate of DDH was not statistically different between those groups (2.5% [0.9%—6.8%] vs. 0.7% [0.2%—2.5%] vs. 1.7% [0.6%—5.3%]; Q (subgroup difference) = 2.363, *p* = 0.307) (Supplementary file 3). DDH incidence was reported higher in Australia (4.3% [1.9%—9.6%]), followed by East Asia (3.5% [1.1%—10.8%]), North America (1.6% [0.1%—21.1%]), and Europe (1.1% [0.4%—3.2%]). However, this difference was not significant (Q (subgroup difference) = 4.192, *p* = 0.241) (Supplementary file 4). Egger’s regression test indicated no publication bias in our main analysis (*p* = 0.07) (Supplementary file 5).

**Table 3:** Incidence of developmental dysplasia of the hip among preterm and term infants. DDH: developmental dysplasia of the hip, SD: standard deviation

Study, year	Preterm definition (Mean week ± SD)	Preterm children (N)		Term definition (Mean weeK ± SD)	Term children (N)		Screening method for DDH	Time of DDH diagnosing	DDH definition based on graf classification
		Event (DDH)	Total		Event (DDH)	Total			
**Gardiner et al., 1990** [14]	24–36 (34)	6	82	37–42 (40)	9	82	Ultrasonography and clinical examination	As soon as possible after birth	≥ IIc
**Xu et al., 2022** [24]	_	33	1716	_	312	18,117	Ultrasonography and clinical examination	_	≥ IIa
**Hegde et al., 2020 **[15]	23–36	112	918	≥ 37	26	226	Ultrasonography and X-ray	At 6 weeks corrected age	≥ IIc
**Koob et al., 2022 **[16]	< 38 (34.9 ± 2.0)	4	283	38–43 (40.2 ± 1.1)	2	377	Ultrasonography	First week after birth	≥ IIb
**Lange et al., 2017 **[17]	< 37 (33 ± 3.4)	3	376	37–42 (39.4 ± 1.2)	42	2534	Ultrasonography	For term infants 3–10 days of age and for preterm infants at the corrected age of more than 36 weeks	≥ IIc
**Orak et al., 2015** [20]	≤ 34 (31.1 ± 2.5)	1	221	40 (40.2 ± 0.3)	1	246	Ultrasonography and clinical examination	First postnatal week	≥ IIc
**Quan et al., 2013** [22]	< 37	3	129	≥ 37	3	163	Ultrasonography and clinical examination	At discharge from hospital and 6 weeks corrected gestational age	Not mentioned
**Pulik et al.,2022** [25]	< 37	2	230	≥ 37	136	2872	Ultrasonography	At 6 weeks of life. In the case of a positive physical examination at birth or risk factors, ultrasound is recommended in the first weeks of life	≥ IIa (-)
**Lee et al., 2016** [18]	< 32 (28.7 ± 2.3), 32- < 37 (34.1 ± 1.3)	20	318	_	_	_	Ultrasonography and clinical examination	_	_
**Sezer et al., 2013 **[8]	< 37 (30.4 ± 2.4)	1	421	_	_	_	Ultrasonography	Third or fourth weeks after delivery	≥ IIc
**Duramaz et al., 2019 **[11]	30–36 (33.0 ± 2.0)	27	394	_	_	_	Ultrasonography	In the first week of their life	≥ IIc
**Jeon et al., 2022 **[6]	< 32	10	155	_	_	_	Ultrasonography	_	_
**Leonard et al.,2022 [**19]	< 35 Median with 25th and 75th quartiles: 32 (29–34)	6	1533	_	_	_	Ultrasonography and clinical examination	Performed at 4 to 6 weeks’ corrected age (defined as 4–6 weeks after the expected term due date)	≥ IIb

### Preterm birth and DDH risk

**Fig. 3:**
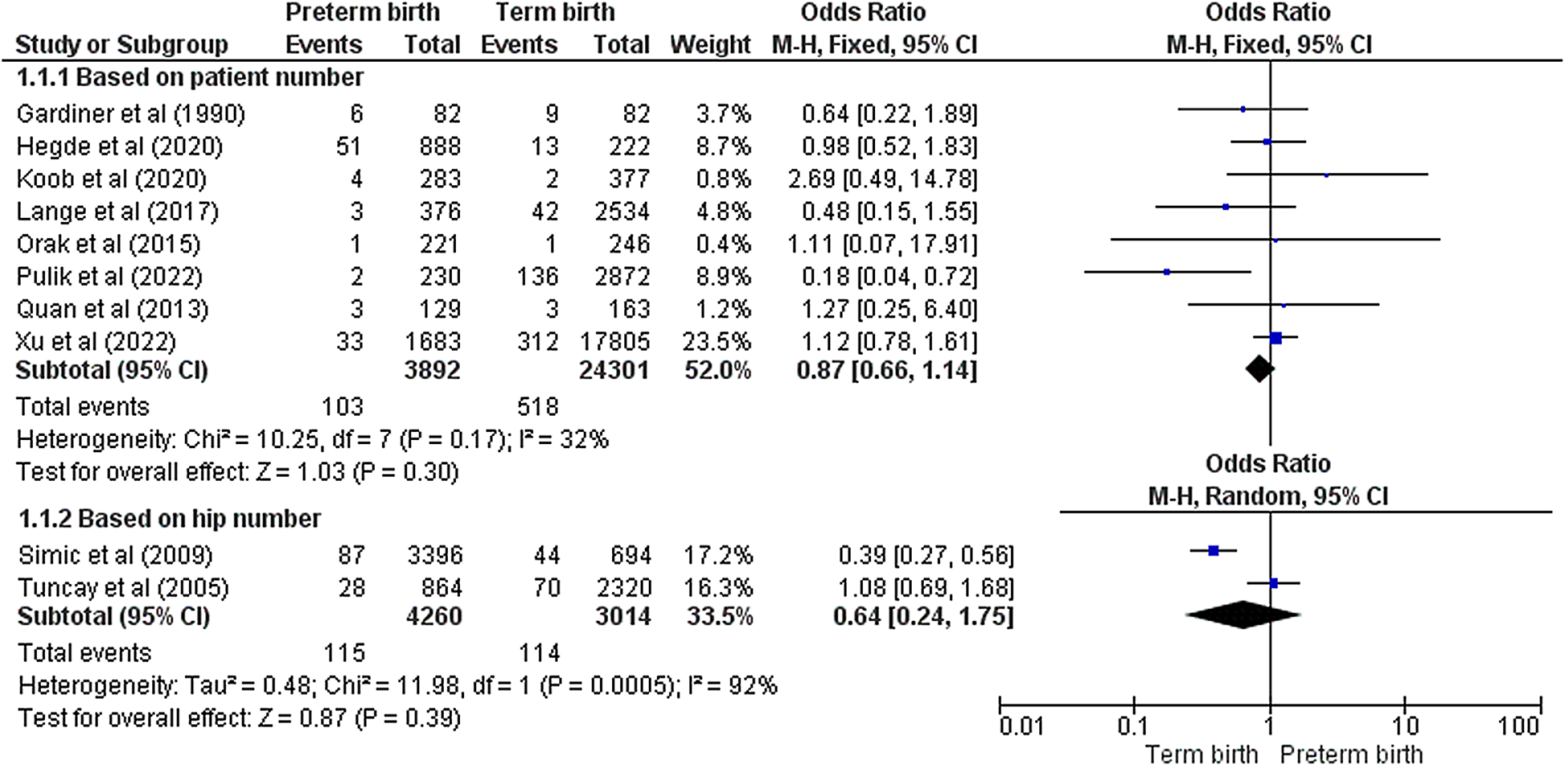
Forest plot of the association between preterm birth and DDH

**Fig. 4:**
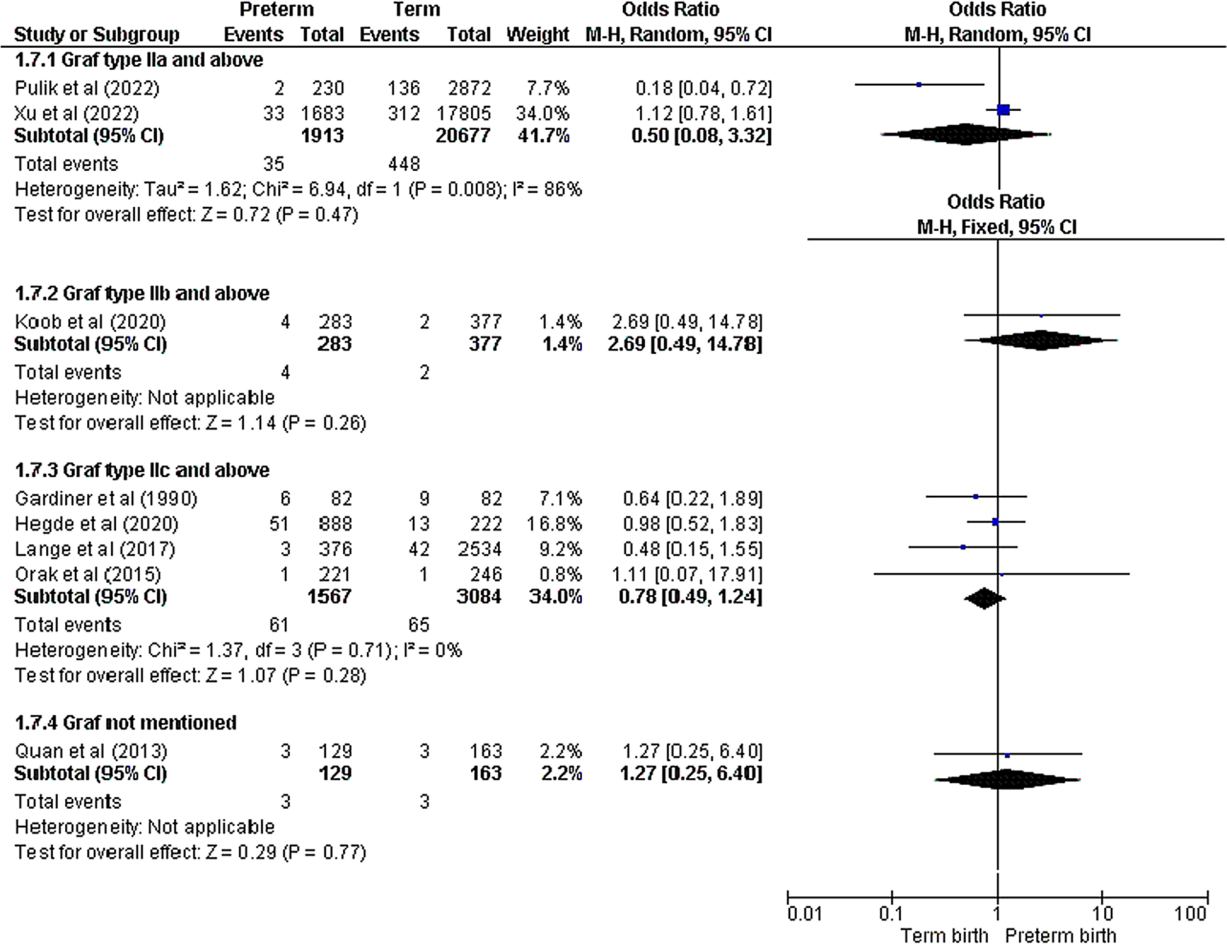
Forest plot of the association between preterm birth and DDH based on Graf types

**Fig. 5:**
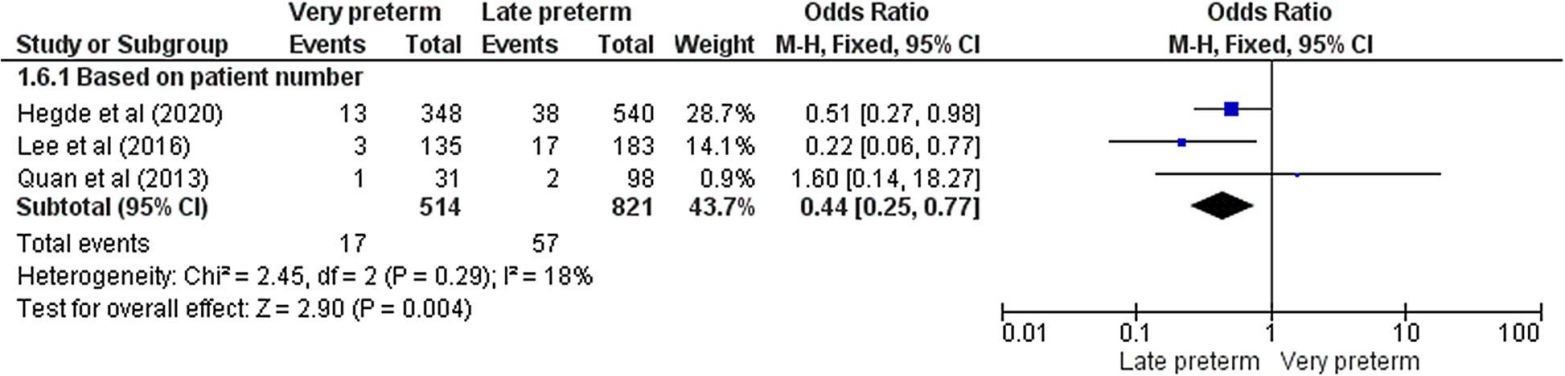
Forest plot of the association between very preterm birth and DDH compared to the moderate to late preterm birth

The major endpoint of our study was to determine whether preterm birth is a risk factor of DDH or not. A total of ten papers reported the proper comparative data. Eight studies reported the number of patients with DDH as their primary outcome [14–17, 20–22, 24]. On the other hand, two other studies considered each hip separately in their analysis [12, 23]. Due to this discrepancy, we decided to analyze these subgroups (patient – hip) separately. The pooled analysis demonstrated that there was no significant difference between preterm and term infants in terms of DDH incidence in either patient subgroup (OR = 0.87 [0.66 – 1.14], Z = 1.03, *p* = 0.30) or hip (OR = 0.64 [0.24 – 1.75], Z = 0.87, *p* = 0.39) (Fig. 3). Low to moderate heterogeneity was noted in patient subgroup (Chi^2^ = 10.25, I^2^ = 32%, *p* = 0.17) unlike the hip subgroup (Chi^2^ = 11.98, I^2^ = 92%, *p* < 0.001). Subgroup analysis based on the DDH definition (according to the Graf types) was also performed utilizing the available data from all eight studies reporting patient number. No meaningful association was observed between preterm birth and DDH in all various definitions (Graf type IIa and above: OR = 0.50 [0.08 – 3.32], Z = 0.72, *p* = 0.47; Graf type IIb and above: OR = 2.69 [0.49 – 14.78], Z = 1.14, *p* = 0.26; Graf type IIc and above: OR = 0.78 [0.49 – 1.24], Z = 1.07, *p* = 0.28; Graf type not mentioned: OR = 1.27 [0.25 – 6.40], Z = 0.29, *p* = 0.77) (Fig. 4). Three studies had sufficient data regarding the DDH risk in very preterm newborns (< 32^nd^ week) compared with those born in 32^nd^ to 37^th^ gestational week [15, 18, 22]. The meta-analysis revealed that very preterm birth was significantly associated with lower DDH incidence (OR = 0.44 [0.25 – 0.77], Z = 2.90, *p* = 0.004) with a low data heterogeneity (Chi^2^ = 2.45, I^2^ = 18%, *p* = 0.29) (Fig. 5).

### Risk factors for DDH in preterm newborns

**Fig. 6:**
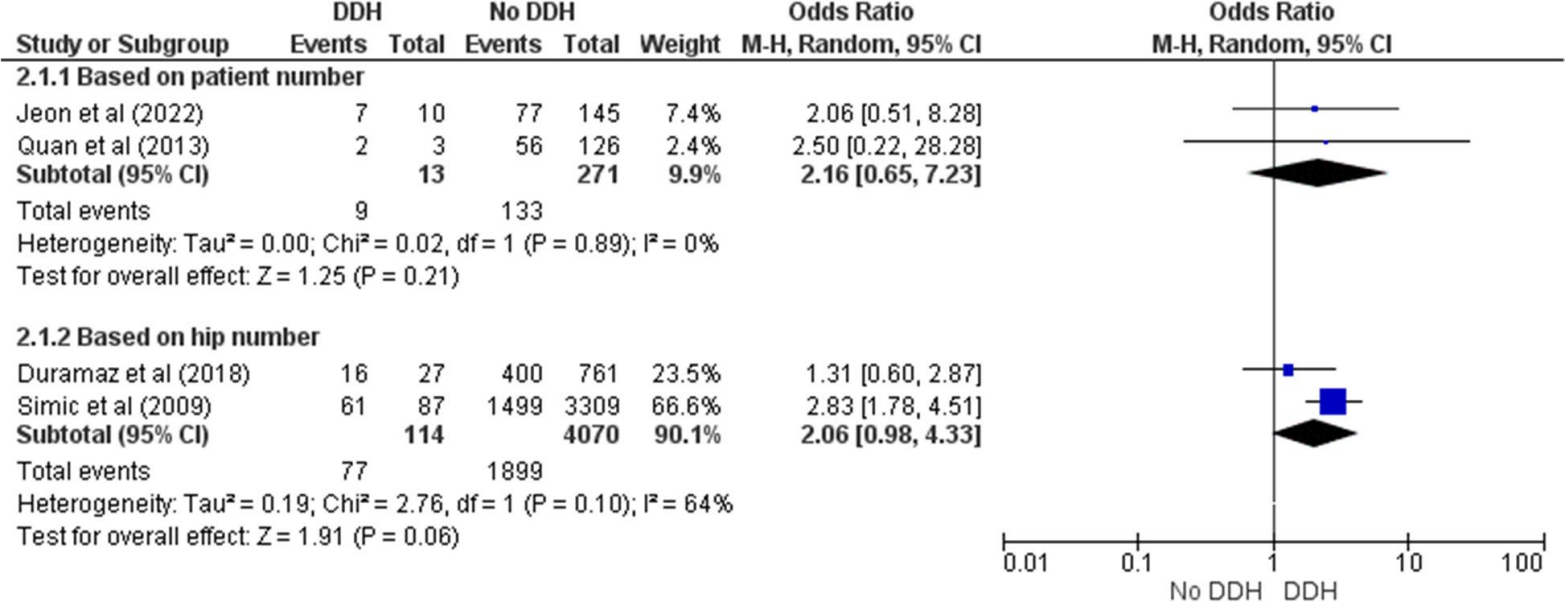
Forest plot of the association between female sex and DDH in preterm newborns

**Fig. 7:**
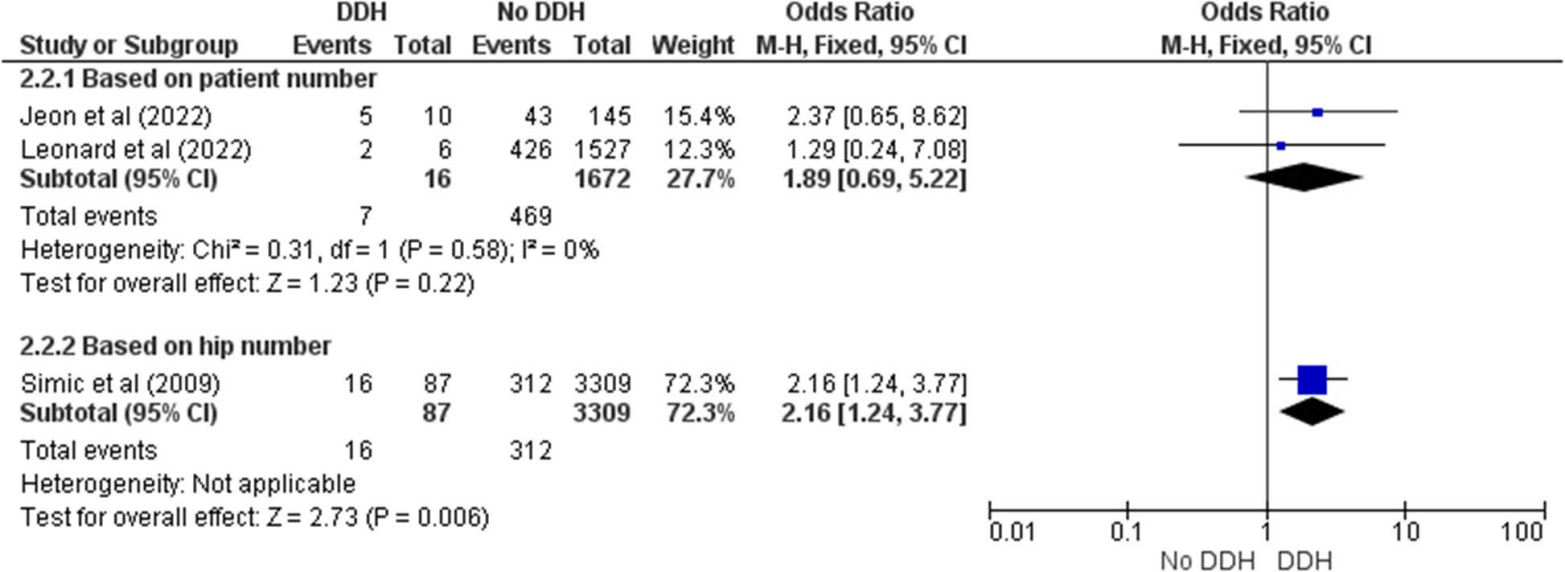
Forest plot of the association between breech presentation and DDH in preterm newborns

Five studies had relevant data about the probable risk factors for DDH in premature infants [6, 11, 19, 22, 23]. Of those factors, only female sex and breech presentation could be quantitatively analyzed. Female sex did not have a significant association with DDH incidence in preterm newborns either in patient-reported (OR = 2.16 [0.65 – 7.23], Z = 1.25, *p* = 0.21) or hip-reported subgroups (OR = 2.06 [0.98 – 4.33], Z = 1.91, *p* = 0.06) (Fig. 6). Simic et al.’s investigation was the only one indicated a significant relationship between sex and DDH (OR = 2.83 [1.78 – 4.51]) [23]. Similarly, there was not a noteworthy association between breech presentation and DDH in patient-reported subgroup (OR = 1.89 [0.69 – 5.22], Z = 1.23, *p* = 0.22) (Fig. 7). However, Simic et al. (reporting hip number) found it statistically significant unlike the others (OR = 2.16 [1.24 – 3.77], Z = 2.73, *p* = 0.006) [23]. Lack of sufficient data concerning other variables did not allow us to perform the meta-analysis. Jeon et al. evaluated some other factors like gestational age and body weight in those treated due to DDH compared to the control group [6]. Nonetheless, none of them was significantly different between two groups (*p* = 0.583, *p* = 0.607, respectively).

## Discussion

Due to the controversy in the literature regarding the association between preterm birth and DDH, we performed this study to systematically review the available data on the topic. Our findings suggest that DDH incidence among preterm neonates is approximately 2 in 100, but the pooled data from the literature did not show a significant association between preterm birth and DDH.

A previous meta-analysis found an incidence of 1.9% for DDH in the general population [26]. We found an incidence of 2% in preterm infants, which does not suggest a higher incidence in these patients. The majority of studies have found geographical differences in the incidence of DDH [27, 28]. We also found a higher incidence in Australia, followed by East Asia, North America, and Europe. It should also be noted that the studies reviewed here are relatively small and were not dedicated to establishing geographical differences. Larger epidemiological studies are needed to confirm the results. In a recent systematic review by Burkhart et al., no association between prematurity and DDH was found [29]. However, the authors only included studies that defined prematurity as birth before 37 weeks of pregnancy, which resulted in a smaller total population.

Although the risk factors for DDH have been previously studied in meticulous meta-analyses, it has not been done in the context of preterm infants. We could extract preterm newborns' data on breech presentation and sex [6, 19, 22, 23]. Sufficient data on other risk factors were either not available or only available in a single study. Regarding both factors, the analysis only found them to be significant influencers on DDH in Simić et al.’s study.

We included a total of 15 articles; however, six could not be included in our primary analysis due to the lack of sufficient statistical information. Our results are also comparable to those of de Hundt et al. regarding prematurity impact on DDH [30]. Four of the studies included in this review found prematurity a protective factor for DDH, while others did not find a significant association [20, 23, 25, 31]. Shorter exposure to maternal hormones and lack of mechanical restrictions in the last weeks of gestation have been proposed as two main possible explanations for this phenomenon [17, 25]. It has been theorized that maternal steroid hormones might have relaxant effects on the fetal hip joint [32, 33]. In addition, preterm newborns are not influenced by some of the intrauterine mechanical problems (luxation-provoking position of the fetus, decreased amniotic fluid, increased fetal size), which are more common in the later stages of fetal development. Hence, their hips may develop unhindered [23]. These might also support our finding of a lower DDH incidence in very premature infants than the moderate-to-late preterm ones.

The definition of DDH was heterogeneous among studies. The definition of preterm birth was also not identical, ranging from 32nd to 37th gestational weeks. Moreover, Simić et al. screened neonates as soon as possible after birth, while others preferred the discharge time or 4–6 weeks of corrected age as their first screening time. These variations in the methods might explain the diversity of results to some degree. Altogether, there is a paucity of data evaluating DDH risk factors in preterm newborns, underpinning the necessity of more robust and conclusive original studies.

Screening for DDH is performed to diagnose and treat patient before complications occur. Different screening protocols are in place throughout the world, from physical examination by primary care physicians to universal ultrasound screening of all newborns [34]. While ultrasound the most sensitive screening method, the costs and resources needed are not to be overlooked [35–39]. Also, ultrasound may result in overdiagnosis and overtreatment of borderline dysplasia or premature hips that would otherwise develop normally [36, 37, 40]. Besides, screening programs come with a great cost for healthcare systems and are not implementable in all settings and populations, so the idea of a more selective instead of universal screening has been used in many countries. Achieving this goal requires a comprehensive assessment of the risk factors involved [41–43]. Based on the results of our study, prematurity may not be an independent risk factor for DDH, and ultrasound screening of otherwise normal preterm infants may not be warranted.

There are limitations to the present systematic review and meta-analysis. First, we could only perform univariate analysis and were not able to explore the combined effects of prematurity and multiple established DDH risk factors due to the lack of. Second, a comprehensive description of our primary variable was not given in a number of articles. Few studies did not report on one or more of these descriptive information; time of diagnosis, term or preterm definition, screening method, or DDH definition. Fourth, there was significant heterogeneity among the included studies in some of our analyses, which were accounted for using a random effects model or subgroup analysis wherever needed. We also reported heterogeneity calculations for all our analyses and calculations so that readers could interpret results more cautiously.

## Conclusions

In a systematic review and meta-analysis of the literature, we did not find preterm birth to be a significant risk factor for DDH. Data suggests that female sex and breech presentation are associated with DDH in preterm infants, as they are in term infants. The findings of this study may help clinicians focus the healthcare resources, including ultrasound screening of the newborns, to patients who are at a greater risk for DDH, and also help policymakers with developing guidelines and screening protocols.

## Supplementary Information

**Additional file 1.**

**Additional file 2.**

**Additional file 3.**

**Additional file 4.**

**Additional file 5.**

## Data Availability

The datasets used and/or analyzed during the current study are available from the corresponding author on reasonable request.

## Abbreviations

DDH: Developmental Dysplasia of the Hip
PRISMA: Preferred Reporting Items for Systematic Reviews and Meta-Analyses
