# Supplementary material for: Preterm birth does not increase the risk of developmental dysplasia of the Hip: a systematic review and meta-analysis
Source: BMC Pediatr. 2023 May 29;23:268. doi: 10.1186/s12887-023-04083-1 (PMC10226229; doi:10.1186/s12887-023-04083-1)
Supplement: Supplementary file 1 — Additional file 1. [file 12887_2023_4083_MOESM1_ESM.docx]

| "Developmental Dysplasia of the Hip"[Mesh]  "Developmental Hip Dysplasia*"[tiab]  "Developmental Dysplasia of the Hip"[tiab]  "Developmental Hip Dislocation*"[tiab]  DDH | AND | "Premature Birth"[Mesh]  "Preterm Birth*"[tiab]  "Premature Birth"[tiab]  "Birth Preterm"[tiab]  "Birth Premature"[tiab]  "Preterm Neonate*"[tiab]  "Preterm"[tiab]  "Premature Infant*"[tiab]  "Preterm Infant*"[tiab]  "Premature" [tiab] |
| --- | --- | --- |

**Pubmed:**

("Developmental Dysplasia of the Hip"[Mesh] OR "Developmental Hip Dysplasia*"[tiab] OR "Developmental Dysplasia of the Hip"[tiab] OR "Developmental Hip Dislocation*"[tiab] OR DDH) AND ("Premature Birth"[Mesh] OR "Preterm Birth*"[tiab] OR "Premature Birth"[tiab] OR "Birth Preterm"[tiab] OR "Birth Premature"[tiab] OR "Preterm Neonate*"[tiab] OR "Preterm"[tiab] OR "Premature Infant*" [tiab] OR "Preterm Infant*" [tiab] OR "Premature" [tiab])

119 results

**Web of Science:**

(TS=("Developmental Hip Dysplasia*"OR "Developmental Dysplasia of the Hip" OR "Developmental Hip Dislocation*" OR DDH) AND TS=("Preterm Birth*" OR "Premature Birth" OR "Birth Preterm" OR "Birth Premature" OR "Preterm Neonate*" OR "Preterm" OR "Premature Infant*" OR "Preterm Infant*" OR "Premature"))

55 results

**Scopus:**

TITLE-ABS-KEY("Developmental Hip Dysplasia*"OR "Developmental Dysplasia of the Hip" OR "Developmental Hip Dislocation*" OR DDH) AND TITLE-ABS-KEY("Preterm Birth*" OR "Premature Birth" OR "Birth Preterm" OR "Birth Premature" OR "Preterm Neonate*" OR "Preterm" OR "Premature Infant*" OR "Preterm Infant*" OR "Premature")

77 results

**Embase:**

#1 'hip dysplasia'/exp OR 'hip dysplasia'

#2 'developmental hip dysplasia':ti,ab,kw OR 'cdh':ti,ab,kw OR 'developmental dysplasia of the hip':ti,ab,kw OR 'developmental hip dislocation':ti,ab,kw OR 'developmental hip dislocations':ti,ab,kw OR 'congenital dislocation of hip':ti,ab,kw OR 'ddh':ti,ab,kw

‘premature labor’

#3 #1 OR #2

#4 'premature labor'/exp

#5 'preterm birth':ti,ab,kw OR 'preterm labor':ti,ab,kw OR 'premature labor':ti,ab,kw OR 'premature birth':ti,ab,kw OR 'birth preterm':ti,ab,kw OR 'birth premature':ti,ab,kw OR 'preterm neonate':ti,ab,kw OR 'preterm neonates':ti,ab,kw OR 'preterm':ti,ab,kw OR 'premature infant':ti,ab,kw OR 'premature infants':ti,ab,kw OR 'preterm infant':ti,ab,kw OR 'preterm infants':ti,ab,kw OR 'prematurity':ti,ab,kw OR 'premature':ti,ab,kw

#6 #4 OR #5

#7 #3 AND #6

487 results
