# Supplementary figures and images for: Preterm birth does not increase the risk of developmental dysplasia of the Hip: a systematic review and meta-analysis
Source: BMC Pediatr. 2023 May 29;23:268. doi: 10.1186/s12887-023-04083-1 (PMC10226229; doi:10.1186/s12887-023-04083-1)

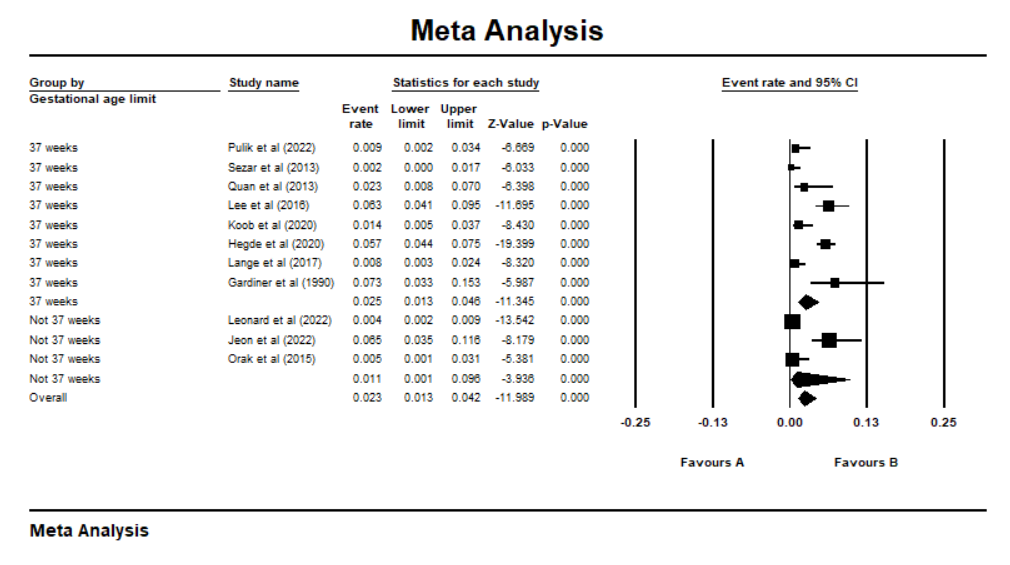

Supplement: Supplementary file 2 — Additional file 2. [file 12887_2023_4083_MOESM2_ESM.png]

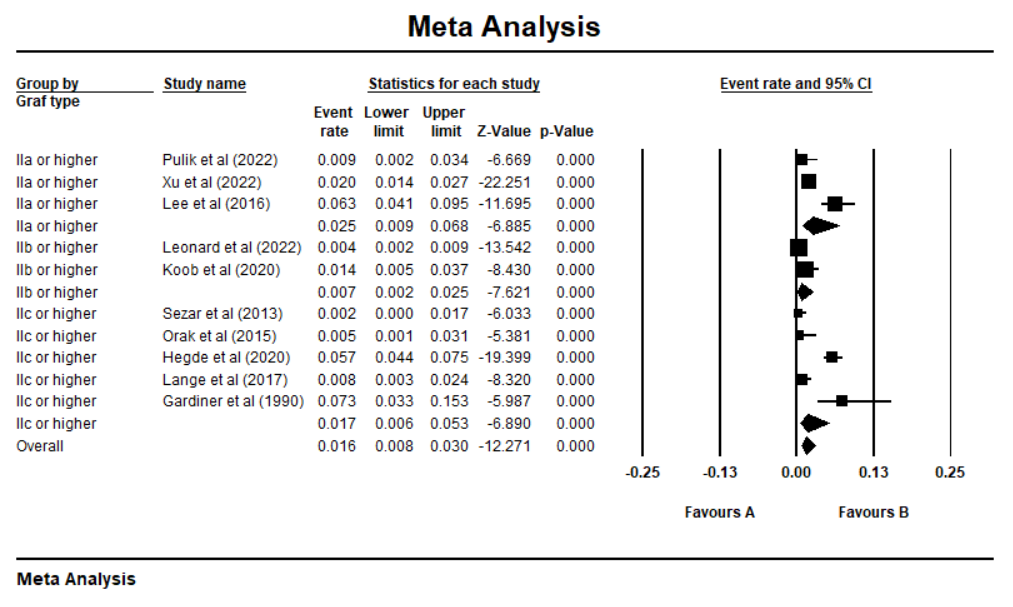

Supplement: Supplementary file 3 — Additional file 3. [file 12887_2023_4083_MOESM3_ESM.png]

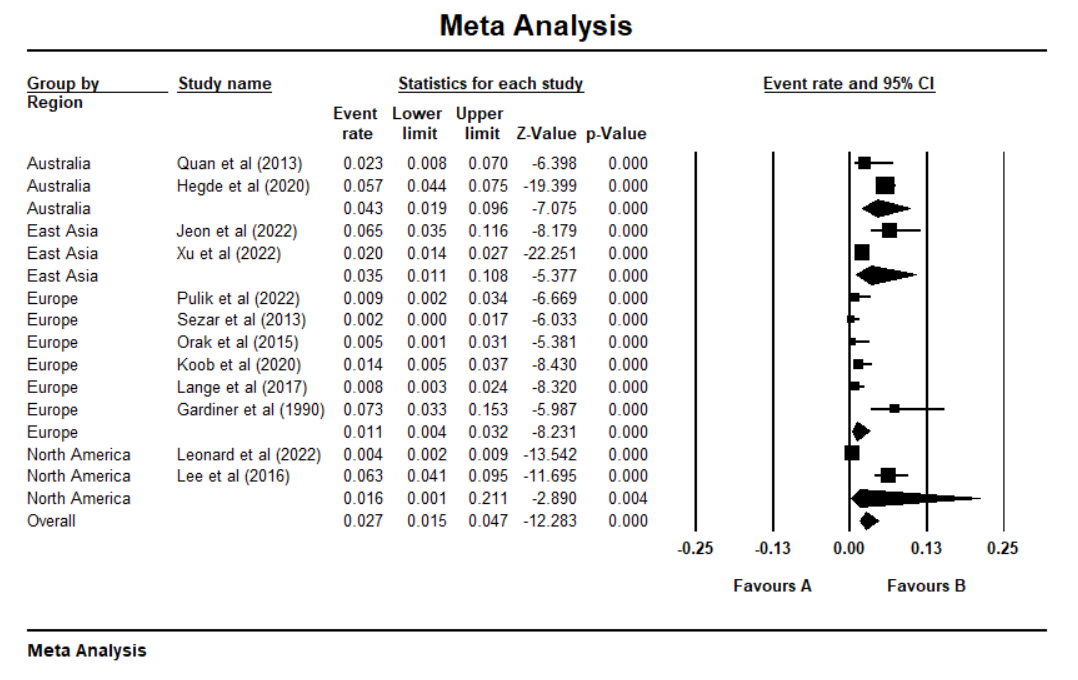

Supplement: Supplementary file 4 — Additional file 4. [file 12887_2023_4083_MOESM4_ESM.png]

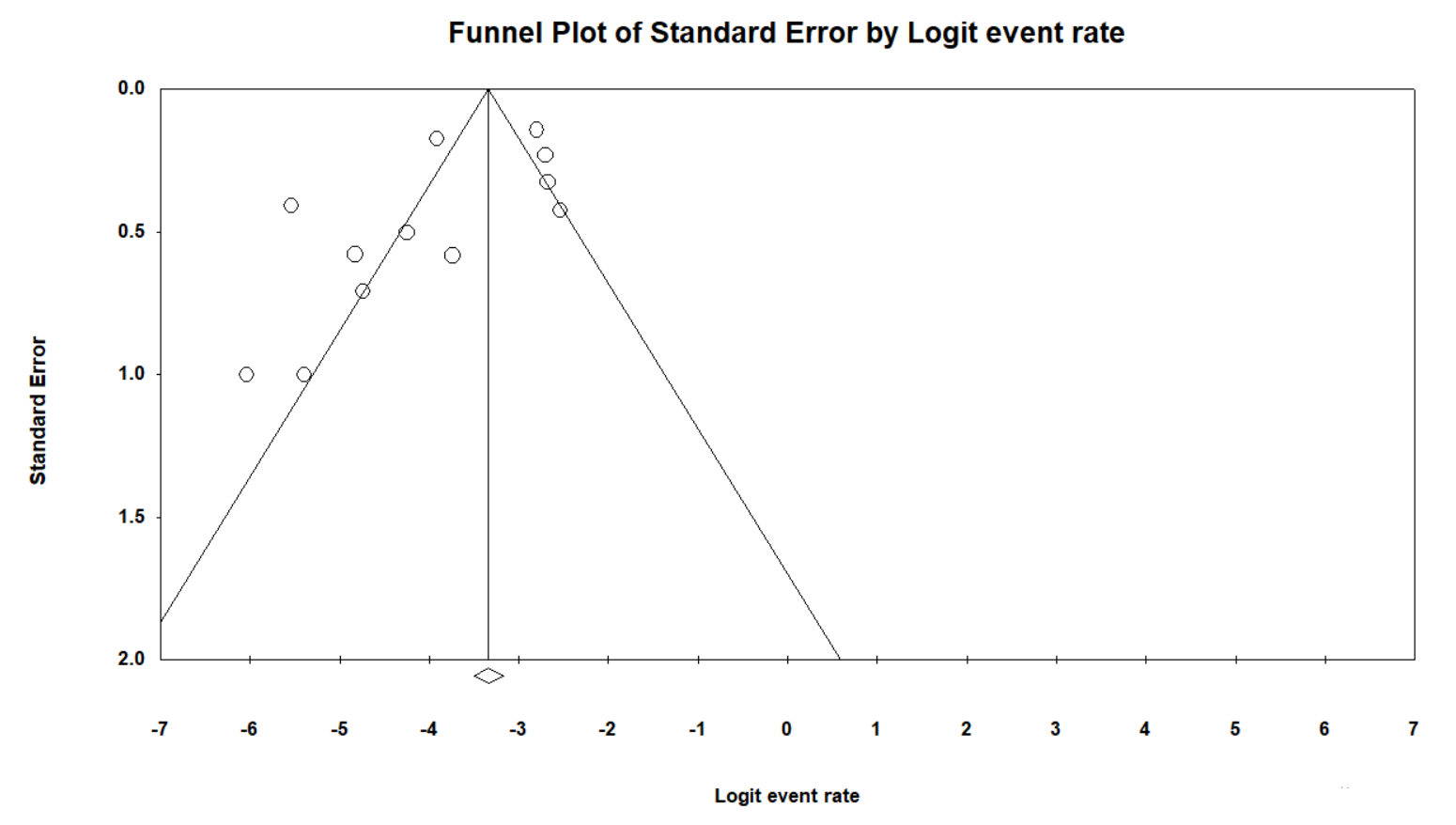

Supplement: Supplementary file 5 — Additional file 5. [file 12887_2023_4083_MOESM5_ESM.png]
